# Clinical value of M1 macrophage-related genes identification in bladder urothelial carcinoma and *in vitro* validation

**DOI:** 10.3389/fgene.2022.1047004

**Published:** 2022-11-16

**Authors:** Yang Yu, Yuexi Huang, Chen Li, Santao Ou, Chaojie Xu, Zhengjun Kang

**Affiliations:** ^1^ The Fifth Affiliated Hospital of Zhengzhou University, Zhengzhou University, Zhengzhou, China; ^2^ Department of Nephrology, The Affiliated Hospital of Southwest Medical University, Luzhou, China; ^3^ Department of Biology, Chemistry, Pharmacy, Free University of Berlin, Berlin, Germany

**Keywords:** M1 macrophage, immunotherapy, chemotherapy, resistance, WGCNA

## Abstract

**Background:** Tumor microenvironment (TME) takes a non-negligible role in the progression and metastasis of bladder urothelial carcinoma (BLCA) and tumor development could be inhibited by macrophage M1 in TME. The role of macrophage M1-related genes in BLCA adjuvant therapy has not been studied well.

**Methods:** CIBERSOR algorithm was applied for identification tumor-infiltrating immune cells (TICs) subtypes of subjects from The Cancer Genome Atlas (TCGA) and Gene Expression Omnibus (GEO) data sets. We identified potential modules of M1 macrophages by weighted gene co-expression network analysis (WGCNA). Nomogram was determined by one-way Cox regression and lasso regression analysis for M1 macrophage genes. The data from GEO are taken to verify the models externally. Kaplan-Meier and receiver operating characteristic (ROC) curves validated prognostic value of M1 macrophage genes. Finally, we divided patients into the low-risk group (LRG) and the high-risk group (HRG) based on the median risk score (RS), and the predictive value of RS in patients with BLCA immunotherapy and chemotherapy was investigated. Bladder cancer (T24, 5637, and BIU-87) and bladder uroepithelial cell line (SV-HUC-1) were used for *in vitro* validation. Reverse transcription-quantitative polymerase chain reaction (RT-qPCR) was employed to validate the associated genes mRNA level.

**Results:** 111 macrophage M1-related genes were identified using WGCNA. RS model containing three prognostically significant M1 macrophage-associated genes (FBXO6, OAS1, and TMEM229B) was formed by multiple Cox analysis, and a polygenic risk model and a comprehensive prognostic line plot was developed. The calibration curve clarified RS was a good predictor of prognosis. Patients in the LRG were more suitable for programmed cell death protein 1 (PD1) and cytotoxic T lymphocyte associate protein-4 (CTLA4) combination immunotherapy. Finally, chemotherapeutic drug models showed patients in the LRG were more sensitive to gemcitabine and mitomycin. RT-qPCR result elucidated the upregulation of FBXO6, TMEM229B, and downregulation of OAS1 in BLCA cell lines.

**Conclusion:** A predictive model based on M1 macrophage-related genes can help guide us in the treatment of BLCA.

## Introduction

Bladder urothelial carcinoma (BLCA) is a common malignant tumor in the bladder system, which was listed as one of the nine most common cancers by the World Health Organization (WHO). Based on histopathology, BLCA can be classified into two categories, muscle-invasive bladder cancer (MIBC) takes most of the BLCA, which is prone to recurrence, another type that accounts for 30% is non-muscle-invasive bladder cancer (NMIBC) with rapid metastasize and low survival rate (Xu et al., 2022a; Kubrak et al., 2022). Therefore, we urgently need a prognostic risk model to provide guidance for the treatment of BLCA.

Tumor microenvironment (TME) is constructed by interacting closely with the extracellular matrix (ECM) and stromal cells to evade detection and eradication by host immune surveillance (Ge and Ding, 2020). Mounting studies have indicated the composition of the TME correlates strongly with immune response and chemotherapy, and regulations in various immune cells from the TME effect the clinical outcome of malignancies (Erbani et al., 2022; Friedrich et al., 2022; Pang et al., 2022; Wang et al., 2022). Tumor-associated macrophages (TAM) drive tumor progression, metastasis, and therapeutic resistance (Larroquette et al., 2022)but heterogeneity is a considerable factor in macrophages (Wu et al., 2020). M1 macrophages inhibit solid tumorigenesis, progression, metastasis, and drug resistance, while M2 macrophages act the opposite. M1 macrophages phagocytose tumor cells *via* cell-mediated cytotoxicity. M1 macrophages exert anti-tumor effects through the production of pro-inflammatory factors (TNF-α, I L-1β, and iNOS), chemokines (CXCL10, CXCL11, and CCL2), antigen-presenting molecules (MHCII), co-stimulatory molecules (CD86, CD80), and antigen-processing peptidases (Jia et al., 2021). In addition, Zeng et al. found M1 infiltration to be a reliable biomarker for predicting prognosis of tumor patients and surpassed biomarkers such as CD8 T cells (Zeng et al., 2020). However, the biological role of M1 macrophages in the prognosis of BLCA has not been well studied (Wu et al., 2022).

Currently, transurethral resection of bladder tumors remains the first-line treatment for patients and is combined with chemotherapeutic agents, but with poor efficacy (Kubrak et al., 2022). Immune checkpoint inhibitors have been proven as a proper option for surgical treatments, however, they merely work in a few types of tumors (Garris et al., 2021; Groeneveld et al., 2021; Barone et al., 2022). Programmed death ligand 1 (PD-L1) expression is the main predictive biomarker for immune checkpoint inhibitor (ICI). Furthermore, tumor mutational burden (TMB) reflects an overall neoantigen load and has the potential to be a predictive biomarker for ICI (Cao et al., 2021a). In summary, we planned to evaluate the sensitivity of different adjuvant treatment modalities for bladder cancer treatment.

Therefore, this study tried to establish an M1 macrophage-based risk score (RS) to comprehensively investigate the sensitivity of the tumor to clinical treatments.

## Materials and methods

### Information collection

We downloaded The Cancer Genome Atlas-Bladder Cancer (TCGA-BLCA) (samples of bladder cancer, *n* = 414; normal tissue, *n* = 19) and GSE31684 (*n* = 93) genetic expression data to obtain sequencing profiles. Clinical data for TCGA-BLCA can also be found in The Cancer Genome Atlas (TCGA) database. A total of 407 tumor samples were uploaded after the missing clinical signs samples were extracted from TCGA database. To further analyze the variations in copy number of BLCA patients, we also obtained somatic mutation data from the TCGA database for BLCA patients to further analyze copy number variation (CNV).

### Distribution of TICs

We obtained the abundance of 22 infiltrating immune cells (TICs) in the TME using the CIBERSORT algorithm on the TCGA-BLCA data of the samples (Zhong et al., 2021).

### Weighted gene co-expression network analysis

The purpose of weighted gene co-expression network analysis (WGCNA) was to find co-expressed gene modules (Cao et al., 2021b). The expression of 16,394 genes in the TCGA-BLCA queue will be used as data, and the result of CIBERSORT will be used as an explanation. To construct an approximate matrix, the soft optimal power (*β*) value is selected by using the pickSoftThreshold function, and the power level with a soft threshold of 1–20 is selected as a candidate. To obtain different gene modules, the tom matrix obtained from genetic expression is used to regroup genes, set the minimum number of modular genes, and cut the outcomes of gene synthesis. By using the “dynamic tree cutting” algorithm, similar genes are introduced into the same module at the same time. Our study targeted “M1 macrophages”, so the modules of most significant relevance to M1 macrophages were selected.

### Construction of prognostic signature in macrophage M1

Using the most important gene module to study the prognosis of M1 macrophage related genes, a prognostic risk signature was constructed in bladder cancer. In the first step, univariate regression analysis was conducted to identify genes that may be important for overall survival (OS). A multivariate Cox regression model was used to determine the final genes to be included after the lasso algorithm. Finally, three M1 macrophage-associated genes were developed, and RS were calculated according to the following equation:

$$riskscore=\overset{n}{\underset{i=1}{\sum }}\left(coefi*Xi\right)$$

Here, *coef* was the regression coefficient in the multivariate Cox regression analysis as described previously. *X* indicated the expression of candidate genes. Where *i* indicated M1 macrophage-associated gene.

### Validation of M1 macrophage-related prognostic features

Each BLCA sample was given a corresponding RS according to the previous risk formula. The cut-off point was set at the median RS. All samples were split into subgroups with low-risk group (LRG) and high-risk group (HRG). First, Kaplan-Meier curves were made to look for differences in prognosis. Moreover, the predictive value was verified by analyzing the time dependence of the receiver operating characteristic (ROC) curves (Xu et al., 2022a).

### Creation and validation of nomogram

To predict overall survival at 1-, 3-, and 5-year, we developed a nomogram combining RS and other clinicopathological features. Finally, we plotted calibration curves capable of showing the prognostic validity of the nomogram.

### Gene Set Enrichment Analysis

The GSEA software was used to look into the function annotation of the c2. cp.kegg.v7.4. symbols and c5. go.v7.4. symbols collections (Cao et al., 2021c). Results with *p*-value < 0.05 were being considered statistically significant. A graph was created based on the first eight outcomes.

### Relationship between the TMB and RS

From the TCGA-BLCA cohort, we got information about somatic mutations. The number of somatic non-synonymous point mutations in each sample was found using the “maftools” R package.

### Correlation of RS with TME

To determine if there was a correlation between RS and TICs, we measured immune cell infiltration in TME using seven different methods including XCELL, TIMER, QUANTISEQ, MCPcounter, EPIC, CIBERSORT, and CIBERSORT-ABS to evaluate the immune infiltrating situation (Xu et al., 2022b). Based on gene expression data, the ESTIMATE algorithm determines how many stromal cells and immune cells are present in a tumor sample using the stromal score and immune score. When you add up the two scores, you get the ESTIMATE score, which can be used to estimate how pure a tumor is. Spearman correlation analysis was used to find a link between RS and TICs.

### Gene set variation analysis

We used the GSVA to estimate the activity of pathways so that we could compare the activity of pathways in different samples, which are listed in the MSigDB database (Xu et al., 2022c).

### Prediction of patient response to immunotherapy

Immune checkpoints have been identified as key places where immune cells can be stopped from working (Zhang et al., 2020). In this study, we looked at how many 47 genes involved in blocking immune checkpoints were expressed in HRG and LRG. Immunophenoscore (IPS) predicts how a tumor will respond to immune checkpoint inhibitor treatment based on how immune-friendly it is. Each immunophenotype (antigen-presenting, effector, suppressor, and checkpoint) is scored by IPS.

### Prediction of the effects of chemotherapy

We made a ridge regression model using the Genomics of Drug Sensitivity in Cancer (GDSC) cell lines and the TCGA gene expression profiles to determine how drug sensitivity is different between HRG and LRG. The half-maximal inhibitory concentrations (IC50) of four chemotherapeutic agents in BLCA patients were calculated using the pRRophetic algorithm.

### Experimental validation

The National Infrastructure of Cells was used to obtain SV-HUC-1 (a line of human bladder epithelial cells) and three lines of human bladder cancer cells (BIU-87, 5637, and T24). F-12K medium was used to grow the SV-HUC-1 cell line. In RPMI-1640 medium, 3 cell lines from people with bladder cancer were grown. All cell lines were kept in an incubator that was set to 37°C and 5% CO_2_. 10% fetal bovine serum and 1% double antibodies were added to all media. Four cell lines were put through reverse transcription-quantitative polymerase chain reaction (RT-qPCR). Three times, the experiment was done the same way. Glyceraldehyde-3-phosphate dehydrogenase (GAPDH) levels were used as an endogenous control. We calculated the relative expression levels of genes FBXO6, OAS1, and TMEM229B using the 2^−ΔΔCt^ method. The sequences of the primers are as follows： FBXO6, 5′-CCC​TAC​CGA​GCT​GTT​GTC​CA-3′ (forward) and 5′- GTT​GAA​CCG​GGG​CAG​GAG​TC-3′ (reverse); OAS1, 5′-AGA​CAC​GTG​TTT​CCG​CAT​GC-3′ (forward) and 5′-GAG​CCA​CCC​TTT​ACC​ACC​TT-3′ (reverse); TMEM229B, 5′- GGAGAATGAGAGGAAGAA -3′ (forward) and 5′- AGAACCAGAACTGATACC -3′ (reverse); and GADPH, 5′-CCT​TCC​GTG​TTC​CTA​CCC-3′ (forward) and 5′-CAA​CCT​GGT​CCT​CAG​TGT​AG-3′ (reverse).

### Statistical analysis

Two groups were compared using Wilcoxon test, and more than two groups were compared using Kruskal Wallis test. Survival curves were analyzed by the Kaplan-Meier log rank test. Spearman analysis was used to determine the correlation coefficient between RS subgroup and somatic mutation frequency. A two-way *p* less than 0.05 was statistically significant. R software was used for all statistical analyses.

## Results

### The TME of BLCA

**FIGURE 1:**
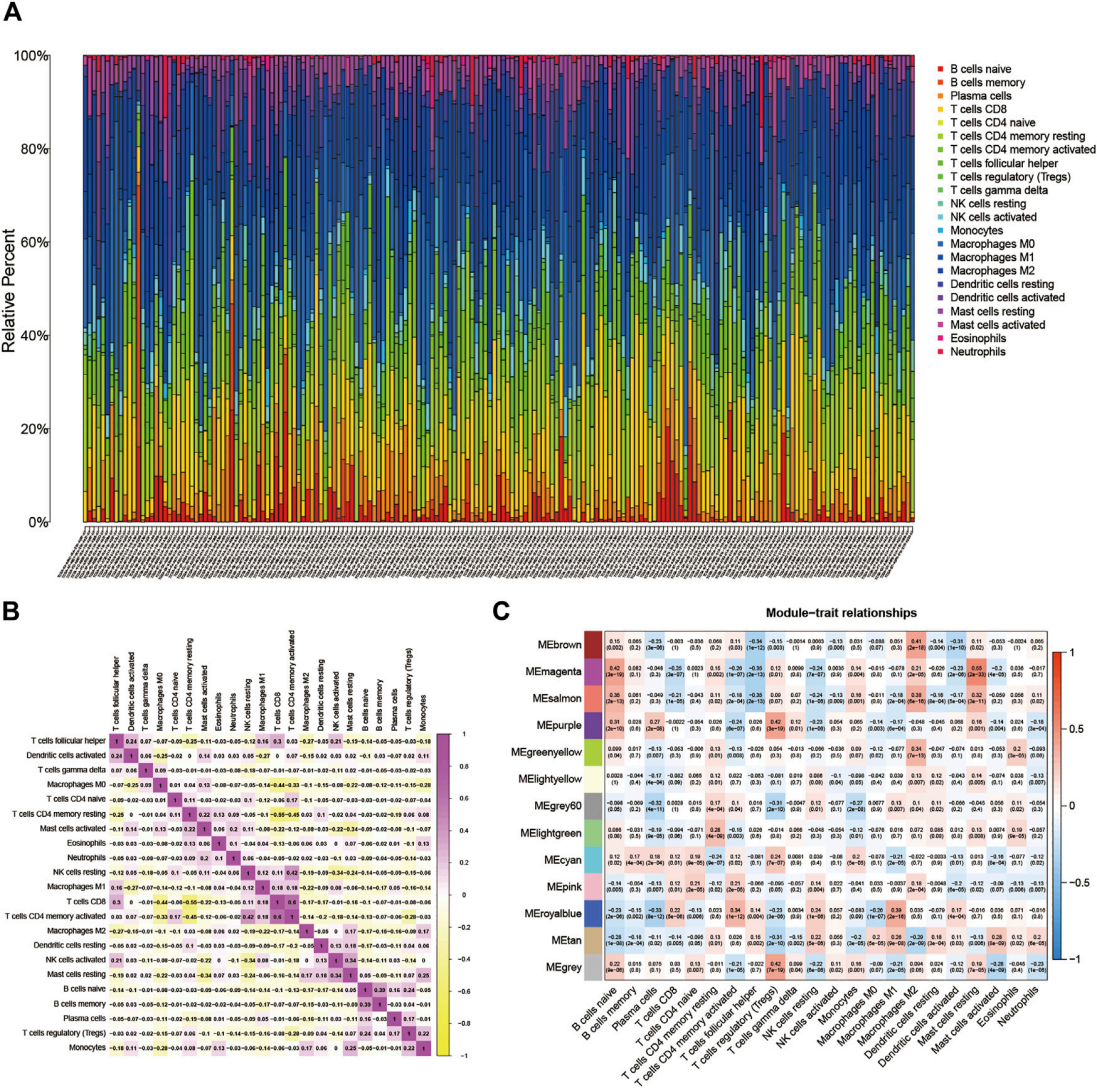
**(A)** Subpopulation of 22 immune cells. **(B)** The intrinsic correlation between 22 immune cells. **(C)** Heatmap of correlation between modules and TICs. Within every square, the number on the top refers to the coefficient between the cell infiltrating level and the corresponding module, and the bottom is the *p*-value.

BLCA’s TME situation is outlined using the CIBERSORT algorithm (Supplementary file: Supplementary Table S1). The 22 TICs in 407 samples are shown in Figure 1A. Supplementary Figure S1A depicts the relationship between the 22 TICs and the clinical phenotype. The potential connections between TICs and the associated relationships are further elucidated in Figure 1B.

### WGCNA network establishment

16,394 permeating gene and TICs sequence documents were analyzed to establish the WGCNA network. First, the optimal soft threshold power (*β*) is set to 9 (Supplementary file: Supplementary Figure S1B). We set the module size to 60, and then inject genes with similar mappings into the same module to construct a hierarchical clustering tree of classes (Supplementary file: Supplementary Figure S1C). Based on the established criteria, the 13 gene modules were grouped and analyzed (using weighting and correlation). In Figure 1C, the horizontal coordinates are shown. There are 22 TIC types, and there are 13 modules in the vertical coordinate. There is a high correlation between the Meroyalblue module and M1 macrophages (cor = 0.39, p = 2e-16). In the present study, we focused on M1 macrophages, so we selected the Meroyalblue module (Supplementary file: Supplementary Table S2) for the follow-up study.

### Establishment of risk signature

**FIGURE 2:**
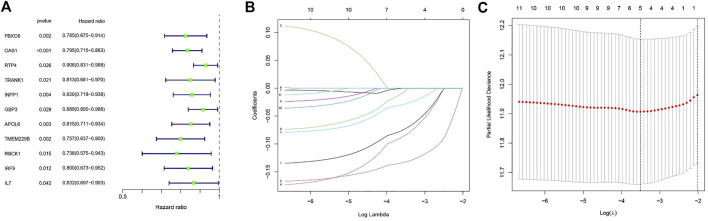
**(A)** Outcomes of univariate cox regression analysis. **(B)** LASSO coefficient profiles of 11 candidate genes. A vertical line is drawn at the value chosen by 10-fold cross-validation. **(C)** The setting parameters selected in the regression process were cross checked ten times. The vertical line represents the final three genes according to the best data.

We extracted clinical information related to bladder cancer from the TCGA-BLCA. Eleven M1 macrophage-associated genes with prognostic value were identified based on univariate Cox analysis (*p* less than 0.05, Supplementary file: Supplementary Table S3), and gene-specific risk ratios are shown in Figure 2A. To prevent overfitting, we performed lasso regression analysis on the screened genes as well as cross-validation to find the optimal values of the penalty parameters. (Figures 2B,C). We then identified three M1 macrophage-associated genes (FBXO6, OAS1, and TMEM229B) as hub genes (all HRs <1, Supplementary file: Supplementary Table S4) by performing multivariate cox regression analysis.

We included three hub genes in the risk profile of BLCA patients and calculated risk scores (RS). Risk score = (−0.1255* expression value of FBXO6) + (−0.1619* expression value of OAS1) + (−0.1507* expression value of TMEM229B). A high-risk group and a low-risk group were determined based on the median cut-off value (0.983).

**FIGURE 3:**
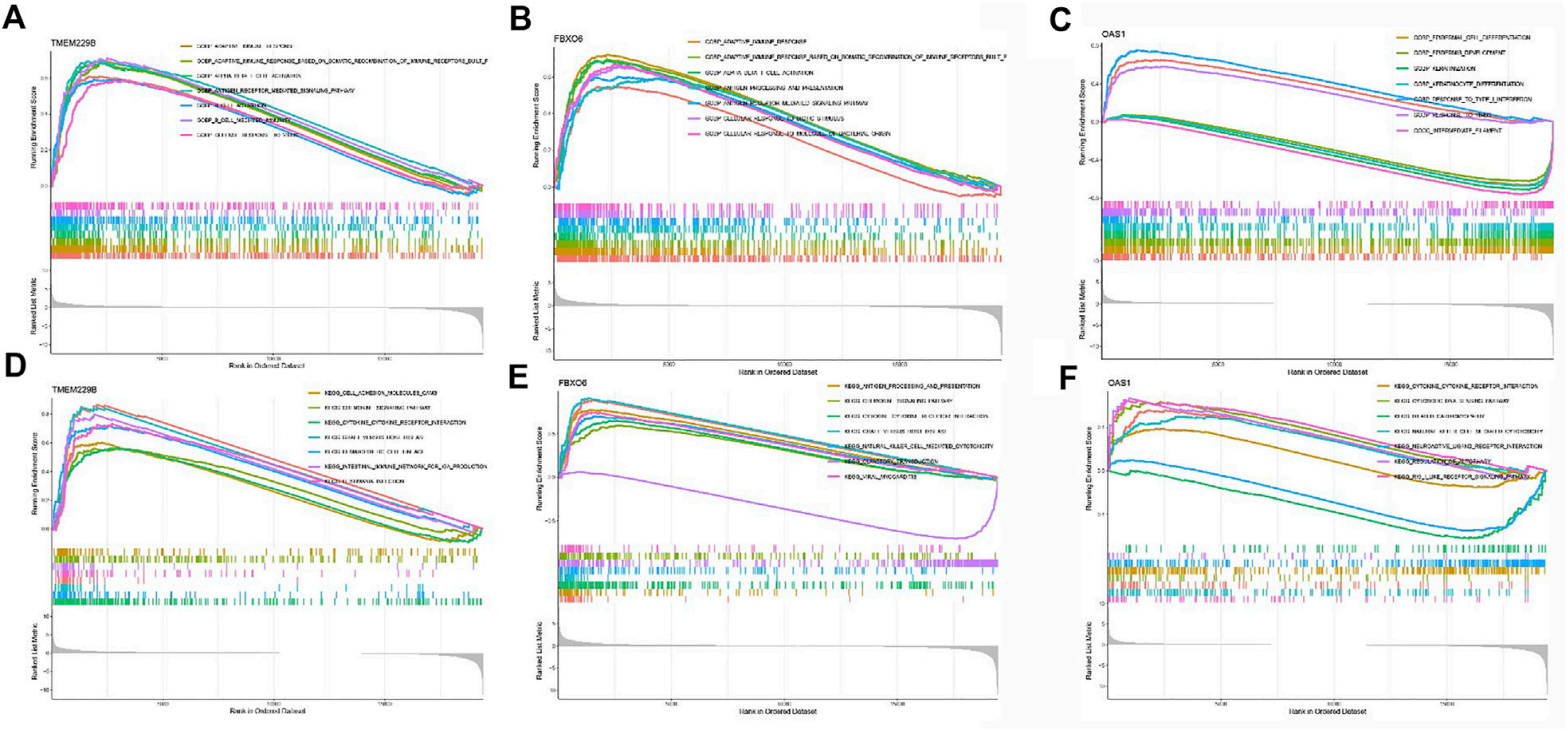
**(A)** The enriched gene sets in GO collection by the high TMEM229 expression sample. **(B)** The enriched gene sets in GO collection by the high FBXO6 expression sample. **(C)** The enriched gene sets in GO collection by the high OAS1 expression sample. **(D)** The enriched gene sets in KEGG collection by the high TMEM229 expression sample. **(E)** The enriched gene sets in KEGG collection by the high FBXO6 expression sample. **(F)** The enriched gene sets in KEGG collection by the high OAS1 expression sample.

Subsequently, GSEA was used to determine the functional enrichment of the hub gene based on the median expression of the hub gene in all samples. Outcomes showed that TMEM229B expression was primarily influenced by *GRAFT* VS*. HOST DISEASE, CELL ADHESION MOLECULES CAMS, CHEMOKINE SIGNALING PATHWAY, HEMATOPOIETIC CELL LINEAGE*, *etc.* (Figures 3A,D). The elevated expression of FBXO6 was mainly associated with *ANTIGEN PROCESSING AND PRESENTATION, CHEMOKINE SIGNALING PATHWAY,* and so on (Figure 3B,E). And the elevated expression of OAS1 was mainly associated with *CYTOSOLIC DNA SENSING PATHWAY, REGULATION OF AUTOPHAG*Y, and so on (Figures 3C,F).

### Validation of risk signature

**FIGURE 4:**
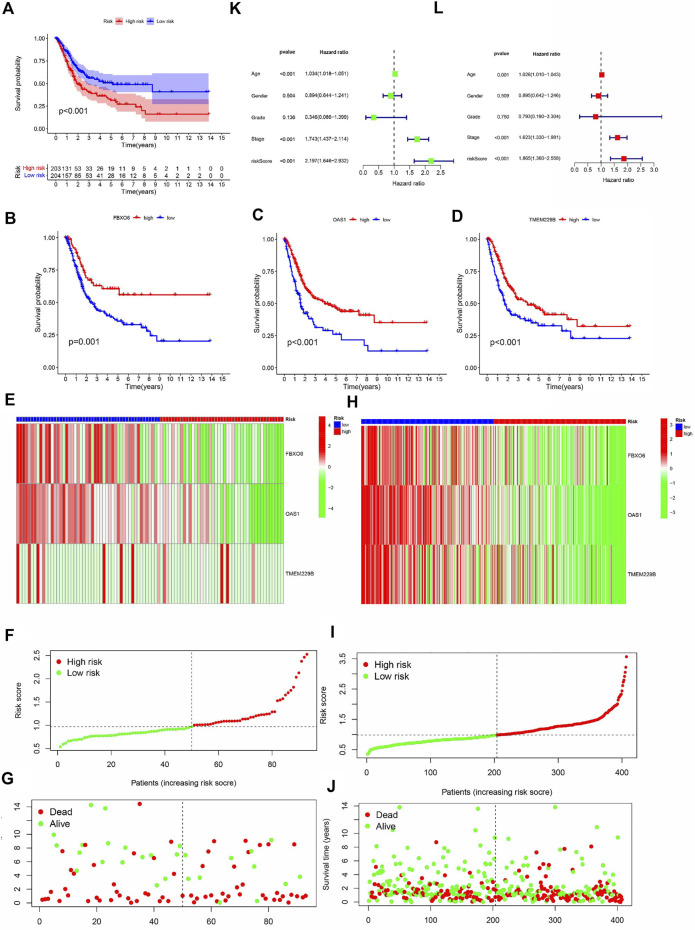
**(A)** The analysis of Kaplan-Meier curve shows that there is a difference in the overall survival rate between high-risk and low-risk groups. Kaplan-Meier curve analysis showed that there were differences in the overall survival rates of FBXO6 **(B)**, OAS1 **(C)**, and tem229b **(D)** in high/low-risk groups. In the GEO **(E)** and TCGA **(H)** cohorts, heat maps of FBXO6, OAS1, and TME229 gene ratios were drawn for each BLCA sample. In the GEO cohort**(F)** as well as in the TCGA cohort **(I)**, the distribution of multi-genes model risk score. In the GEO cohort**(G)** as well as in the TCGA cohort**(J)**, the survival status and duration of BLAC patients.

Kaplan-Meier curves showed that OS was lower in the HRG than in the LRG (*p* < 0.001) (Figure 4A), and OS was lower in the FBXO6, OAS1, and TMEM229B low-expression groups than in the high-expression group (*p* = 0.001, *p* < 0.001, *p* < 0.001) (Figures 4B–D). In the TCGA cohort, the expression of FBXO6, OAS1, and TMEM229B gradually decreased with increasing RS. And the point distribution of RS and survival status indicated that BLCA patients in the low-risk group had a longer OS than the HRG, also in the GEO cohort (Figures 4E–J). Univariate Cox analysis showed that the RS hazard ratios (HR) was 2.197 (95% of confidence interval 1.642–2.932) (Figure 4K), and the multivariate Cox regression analysis showed that the RS was 1.865 (95% of confidence interval 1.360–2.558) (Figure 4L).

To objectively evaluate the performance of our novel M1 macrophage signature, our studied signature was compared with traditional M1 macrophage markers (CD80, TNF-α, and iNOS) (Supplementary Figures S2B, S2F). Further, we combined the novel and traditional M1 macrophage signatures and found that the integrated signature had comparable performance to the novel signature we studied (Supplementary Figures S2A, S2F). T cells in the tumor microenvironment are essential bladder cancer immunotherapy-associated cells. We analyzed the correlation of the gene expression of T cell marker signature (CD4, CD8A, CCR4) and inhibitory molecule signature (CD279, CTLA4, HAVCR2) in HRG/LRG, and the results were shown in Supplementary Figure S2H. Our novel M1 macrophage marker signature was also compared with T cell marker signature and inhibitory molecule signature with ROC curves and C index at 1, 3 and 5 years as shown in Supplementary Figure S2C-E and Supplementary Figure S2G. In conclusion, RS can be used as an indicator to assess the prognosis of BLCA.

### Risk signature and clinicopathological variables

**FIGURE 5:**
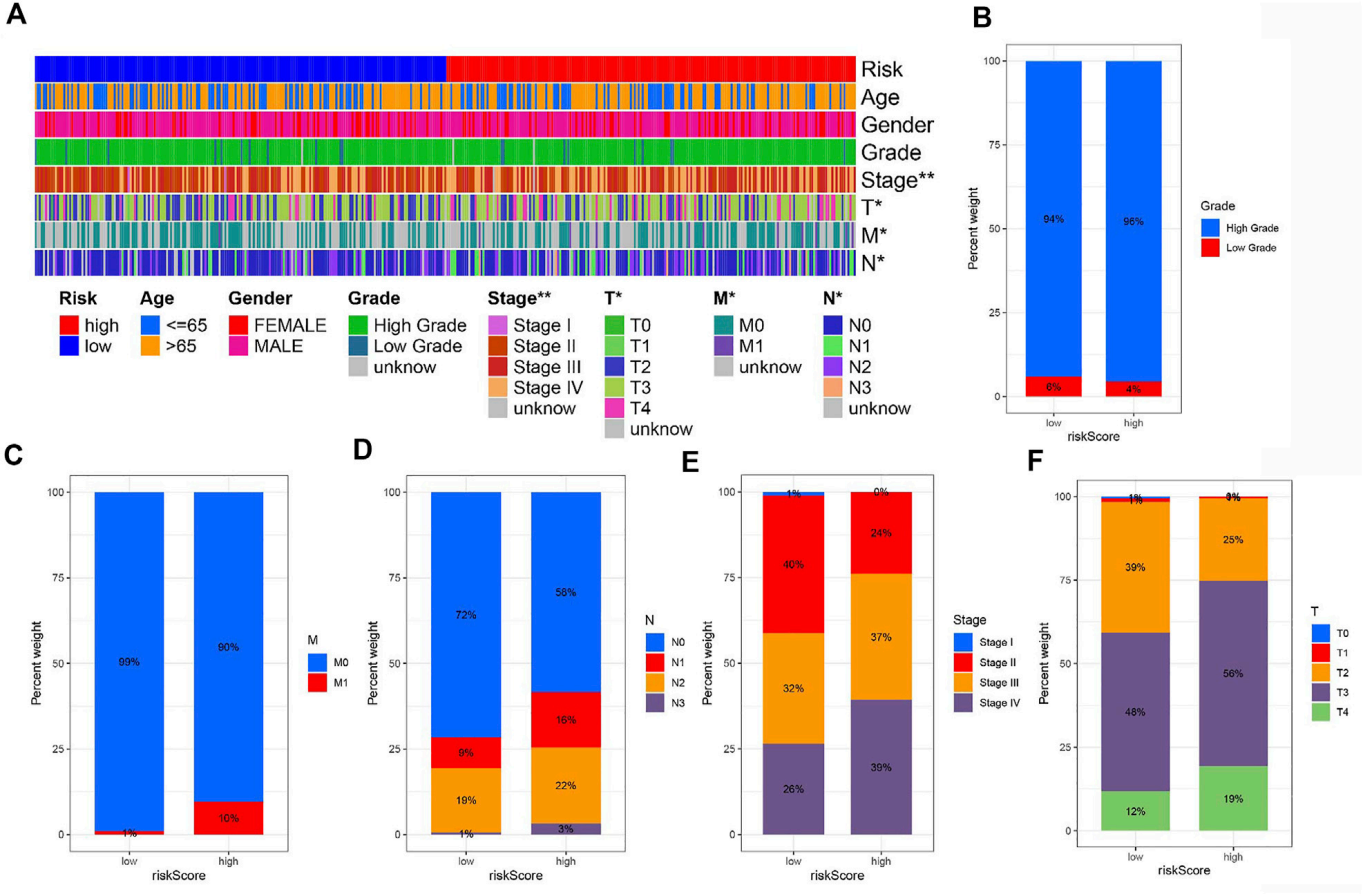
**(A)** The heat map shows the distribution of clinical characteristics and the corresponding risk score proportion in each sample. Rate of clinical variables subtypes in high or low risk score groups. **(B)** Grade, **(C)** M status, **(D)** N status, **(E)** Stage and **(F)** T status.

To explore the correlation of risk and clinicopathological variables, we visualized a plot based on clinicopathological features. Figure 5A displays the distribution of clinical variables in HRG/LRG. There was different in grade, stage, T stage, N stage, and M stage (Figures 5B–F). The results were consistent with clinical practice. These findings, combined with results of univariable and multivariable regression analysis, emphasized that our risk score was indeed good prognostic predictive indicator independent from other clinical parameters.

### Creation and validation of nomogram

**FIGURE 6:**
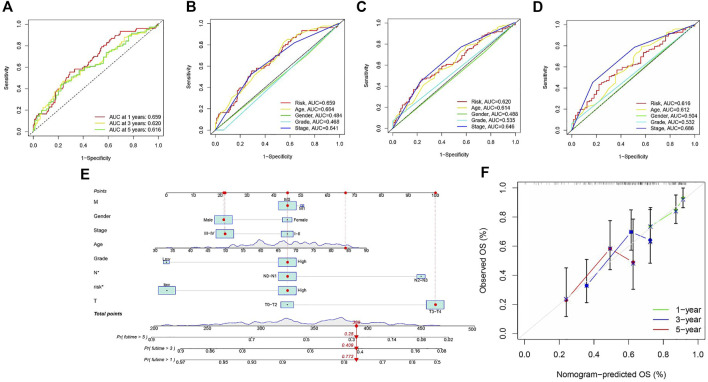
**(A)** ROC analysis is utilized to estimate the prediction characteristics of the prediction. **(B–D)** The prediction area under the risk assessment curve has other clinical characteristics of 1, 3, and 5-year total survival time. **(E)** Nomograms composed of clinical variables and risk characteristics were used to predict the survival rate of BLCA patients. **(F)** Calibration curves of 1-, 3-, and 5-year nomograms.

Area under the Curve (AUC) of ROC of BLCA patients is 0.659, 0.620, and 0.616, respectively, which indicates high traceability for RS (Figure 6A). Next, we combined RS, age, gender, tumor grade, and clinical stage were then combined to analyze AUC for 1, 3, and 5 years. RS’s AUC value was higher (Figures 6B–D), which further proved that RS had a better prognostic value. There is a nomogram consisting of RS, age, gender, tumor grade, clinical stage, T status, M category, and N category for quantitative prediction (Figure 6E). A good prediction performance is indicated by Figure 6F, indicating that the prediction nomogram was accurate.

### TMB and prognosis

**FIGURE 7:**
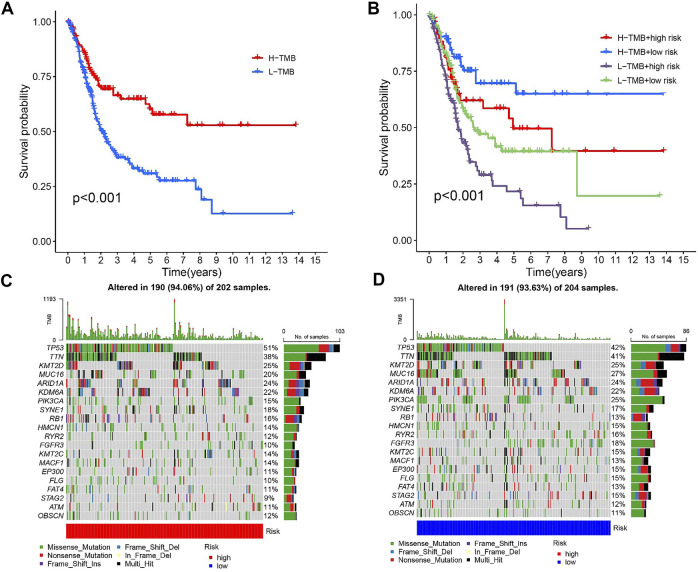
**(A)** Kaplan-Meier curve of high-level TMB group and low-level TMB group. **(B)** Kaplan-Meier curve is used to classify the risk level according to TMB. **(C)** The oncoPrint was constructed using a high risk score. **(D)** The oncoPrint was constructed using a low risk score.

Previous studies have shown that patients with high TMB respond significantly better to immunotherapy (Samstein et al., 2019). Due to this, TMB has become one of the most important biological references for predicting tumor behavior and immunotherapy response. In this study, we found that the overall survival time was significantly lower for high TMB values (*p* < 0.001, Figure 7A). TMB and RS data were used to divide the patients into four subgroups. Patients with low TMB/high RS had the worst prognosis according to the survival analysis. (*p* less than 0.001, Figure 7B). The above outcomes demonstrate that TMB has an impact on the prognosis of BLCA. In addition, we further described the distribution of gene somatic mutations in the HRG/LRG (Figures 7C,D).

### Risk signature in tumor immune microenvironment context of BLCA

**FIGURE 8:**
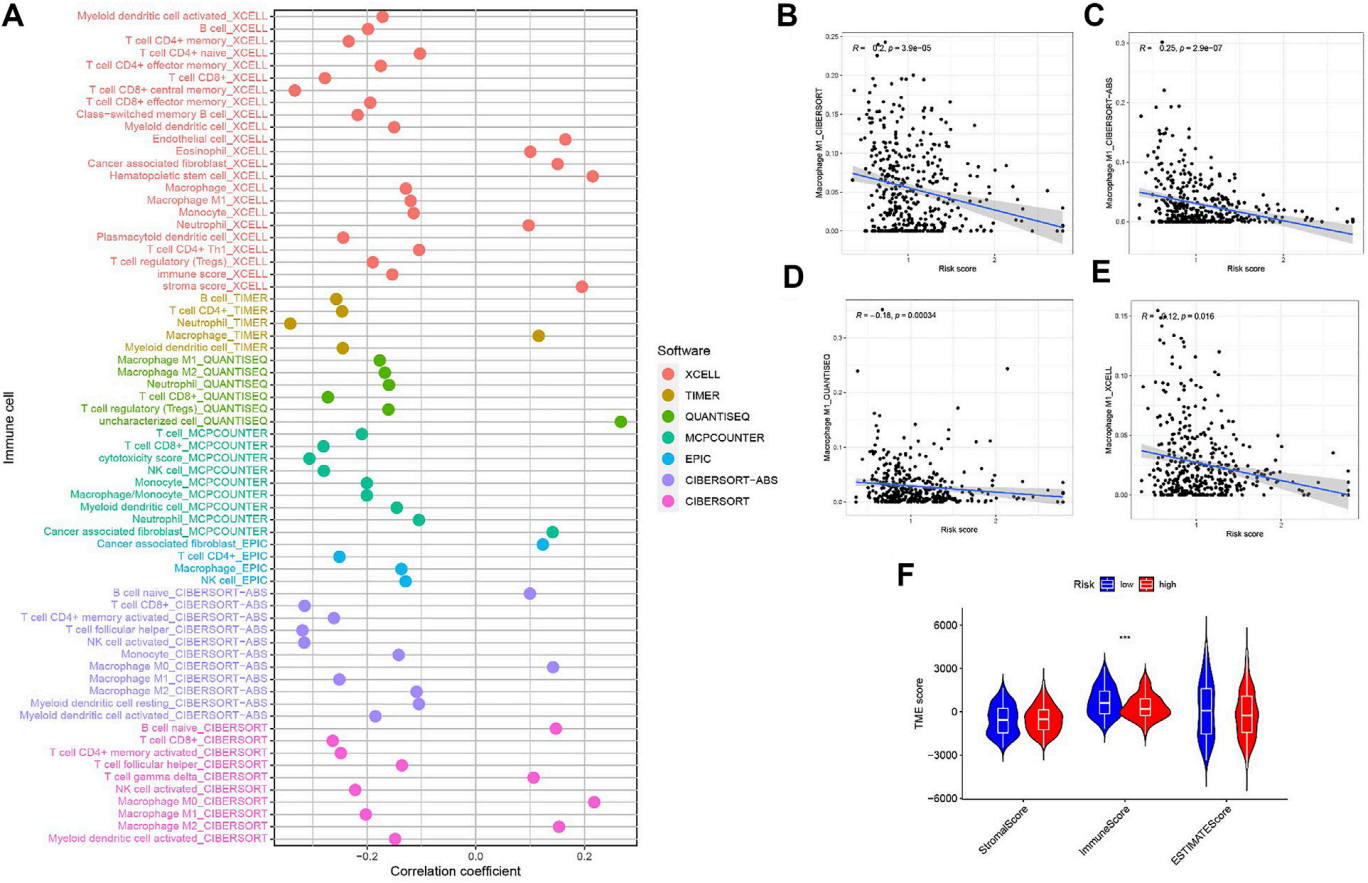
**(A)** Patients in the high-risk group were more positively associated with tumor-infiltrating immune cells, as shown by Spearman correlation analysis. **(B–E)** Correlation analysis of M1 macrophages and risk scores. **(F)** Correlation between TME score with risk scores.

Since M1 macrophages-based risk score and infiltration immune cells had intrinsic and intimate connection, we further explored the potential contribution of risk score in complexity and diversity of tumor immune microenvironment. Infiltration of immune cells and RS are correlated, as is shown in Figure 8A. Outcomes can be found in Supplementary file: Supplementary Table S5. Figures 8B–E reveals the correlation between M1 macrophages and risk score, suggesting a negative correlation between M1 macrophages and RS. Figure 8F shows that the immune score tends to be significantly higher in LRG.

### Signaling pathways in two different risk groups

**FIGURE 9:**
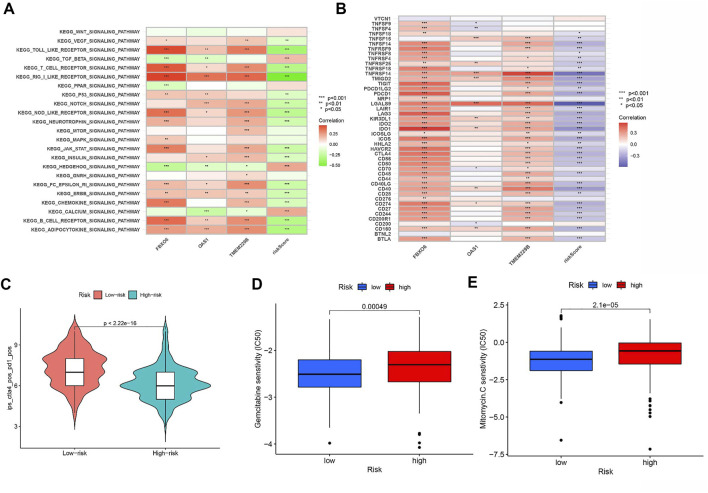
**(A)** Heatmap showing the correlation of representative pathway terms of KEGG with risk score. **(B)** Correlation of expression level of immune checkpoint blockade genes with risk score. **(C)** IPS score distribution plot. Estimation of risk score of chemotherapeutic effect. **(D)** Sensitivity analysis of Gemcitabine in patients with high and low risk score. **(E)** Sensitivity analysis of Mitomycin C in patients with high and low risk score.

We further revealed the biological roles of signaling pathways in tumorigenesis and development in different risk groups by performing GSVA. Figure 9A shows that *TGF BETA SIGNALING PATHWAY, HEDGEHOG SIGNALING PATHWAY, and CALCIUM SIGNALING PATHWAY* activities were enhanced in the HRG.

### Predicting clinical outcomes of immunotherapy in BLCA patients

Most genes associated with immune checkpoint blockade were found to be significantly negatively associated with risk scores (Figure 9B). According to the risk assessment system, LRG has a high IPS score (PD1-positive, CTLA4-positive, Figure 9C). Thus, PD1 and CTLA4 combined immunotherapy is suitable for LRG patients. While patients with HRG were more suitable for novel immunotherapies. The above outcomes suggest that RS correlates with immunotherapy response and that RS helps predict prognosis.

### Prediction of chemotherapy response

We estimated the IC50 of two chemotherapeutic agents (gemcitabine and mitomycin C) in patients with BLCA according to the pRRophetic algorithm, which exhibited a higher IC50 in patients with HRG (both *p* < 0.05; Figures 9D,E). The results revealed that patients with LRG were more sensitive to chemotherapeutic agents.

### Detection of mRNA levels of hub genes by RT-qPCR

**FIGURE 10:**
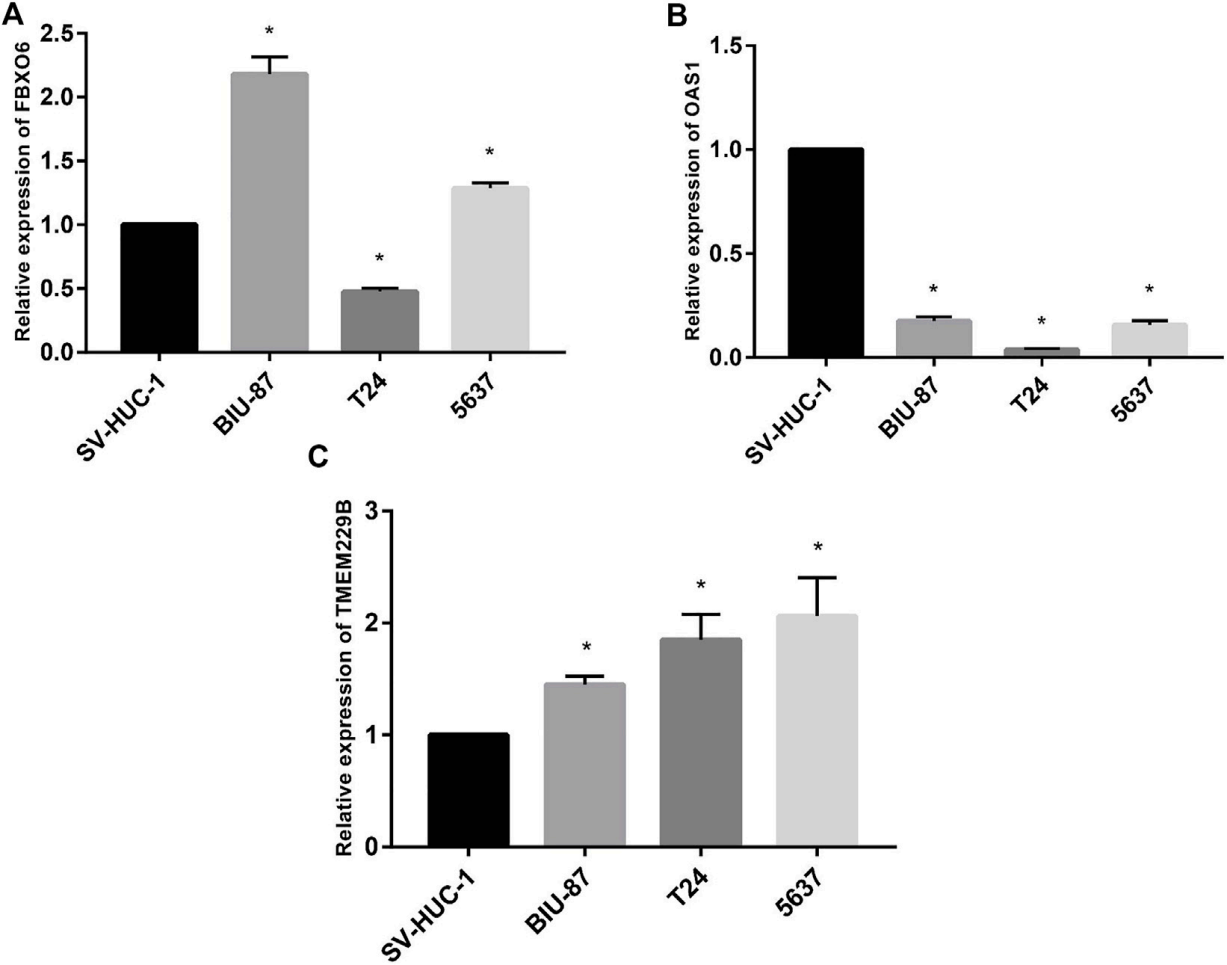
**(A)** The mRNA level of FBXO6 in normal urothelial cell line (SV-HUC-1) and three BLCA cell lines (BIU-87, T24, and 5637) was analyzed by RT-qPCR. **(B)** The mRNA level of OAS1 in normal urothelial cell line (SV-HUC-1) and three BLCA cell lines (BIU-87, T24, and 5637) was analyzed by RT-qPCR. **(C)** The mRNA level of TMEM229B in normal urothelial cell line (SV-HUC-1) and three BLCA cell lines (BIU-87, T24, and 5637) was analyzed by RT-qPCR.

To test the study hypothesis, in human bladder epithelial cells, we detected the expression of FBXO6, OAS1, and TMEM229B genes, as well as three different bladder cancer cell lines by using RT-qPCR technique. OAS1 values were significantly lower in normal bladder epithelial cells than in bladder cancer cells (Figure 10B). Contrary to bladder epithelial cells, bladder cancer cells expressed significantly higher levels of FBXO6 and TMEM229B genes (Figures Figure10A,C). RT-qPCR outcomes well supported our findings.

## Discussion

BLCA is one of the most common and most aggressive malignancies, the worried is that little progress has been made in the treatment of BLCA in the last few decades (Kimura et al., 2020; Lv et al., 2020). It is worth noticing that immunotherapy has shown great potential to treat MIBC and metastatic bladder cancer in the clinic (Nair et al., 2020; Pfail et al., 2021; Zhang et al., 2022). Previous studies have found that macrophages exert different effects on immunotherapeutic responses in advanced cancers (Petty and Yang, 2017; DeNardo and Ruffell, 2019; Zeng et al., 2020; Leblond et al., 2021). Sun et al. (Sun et al., 2022) found that the infiltration and polarization status of TAMs can predict outcomes of survival and chemotherapy benefits, as well as immunotherapy sensitivity in MIBC. Another study reported the value of M1 macrophages as a predictive biomarker for ICI treatment in patients with metastatic uroepithelial carcinoma (Zeng et al., 2020). These suggest that M1 macrophages take an irreplaceable role in tumor development.

In the current work, we obtained a total of 407 BLCA samples and 16,394 genes from the TCGA-BLCA and GSE31684 datasets to further study. The abundances of 22 TICs were obtained from the CIBERSORT algorithm, and the Meroyalblue module highly associated with M1 macrophages was constructed by WGCNA. Subsequently, Cox regression analysis was performed to identify three hub genes and took three hub genes in the risk profile of BLCA patients to calculate the RS. We also proved that RS can predict independently by Kaplan-Meier analysis and regression analysis. The nomogram was built and clinicopathological variables were investigated to strengthen the model prognostic value.

Our prognostic model was built based on three novel M1 macrophage-associated genes (FBXO6, OAS1, and TMEM229B). FBXO6 is an important member of the F-box protein family containing the FBA structural domain, which targets the DNA damage checkpoint kinase Chk1 to destroy S-phase arrest cells, phosphorylates during mitosis and dephosphorylates cells upon entry into the G1 phase. OAS1 is an interferon-induced protein that synthesizes adenosine oligomers from ATP to prevent tumor growth and cell differentiation (Piran et al., 2021). The TMEM is a family of proteins that span biological membranes. Many studies showed that the TMEM family can be described as tumor suppressors or oncogenes, and TMEM229B was reported can be a potential antigen for esophageal squamous cell carcinoma mRNA vaccines (Lu et al., 2022).

GSEA functional enrichment indicated that high expression of FBXO6 is related to immune response and chemokine signaling pathway, the elevated expression of OAS1 was associated with regulation of autophagy, and the elevated expression of TMEM229B was associated with humoral immune response. These outcomes suggest that the three hub genes are widely involved in tumor immunity, which provides a basis for our subsequent assessment of the efficacy of immunotherapy for RS.

TMB can predict survival after immunotherapy in types of cancer, especially when PD-1/PD-L1 is blocked. Moreover, TMB is a promising biomarker of the immune response. TMB may be an independent prognostic factor for multiple cancer immune responses. Therefore, we selected TMB as a prognostic indicator in this study and found patients with a high incidence rate of bladder cancer fared better than before. Subsequent stratified curves of survival showed that patients with low TMB/high RS had the worst prognosis, as well as that risk scores had independent prognostic predictive power.

Macrophages are important members of the innate immune response, and the polarization of macrophages allows them to have diverse functions. Their polarization status depends on environmental changes (Locati et al., 2020; Boutilier and SJIjoms, 2021). Direct metabolism of metabolites or cytokines drives the plasticity and heterogeneity of the tumor microenvironment (Mehla and Singh, 2019). We found that risk score was negatively associated with immune activating cell subpopulations such as CD8 T cells, and M1 macrophages were also negatively associated with RS. And immune scores were relatively low in the HRG. In summary, we speculated that the LRG group may be in immune activation state, which suppresses tumor progression and improves prognosis. In addition, TGFβ signaling pathway, *HEDGEHOG SIGNALING PATHWAY*, and *CALCIUM SIGNALING PATHWAY* were also activated in high-risk groups, suggesting that the high-risk group has different molecular mechanisms.

Finally, we explored the predictive value of RS in BLCA immunotherapy and chemotherapy. The results show that ICB-related genes had a significantly negative risk score, which indicates a higher IPS score for LRG. This suggests that LRG patients are suitable for PD1 and CTLA4 combination with immune therapy, whereas HRG patients prefer to novel immunotherapies.

There are still some limitations in our study, the functions of M1 macrophage-related genes need to be further explored in animal experiments. More importantly, we need to further validate the prediction model in different cohorts collected by multiple centers.

## Conclusion

In conclusion, the RS model based on M1 macrophages can be utilized to predict clinical outcomes, treatment outcomes, as well as prognosis of BLCA patients and provide reference for the treatment of BLCA somehow.

## Data Availability

The datasets presented in this study can be found in online repositories. The names of the repository/repositories and accession number(s) can be found in the article/Supplementary Material.

## Glossary

TME: tumor microenvironment
BLCA: bladder urothelial carcinoma
TICs: tumor-infiltrating immune cells
TCGA: Cancer Genome Atlas
GEO: Gene Expression Omnibus
WGCNA: weighted gene co-expression network analysis
ROC: the receiver operating characteristic
LRG: low-risk group
HRG: high-risk group
RS: risk score
RT-qPCR: reverse transcription-quantitative polymerase chain reaction
PD1: programmed cell death protein 1
CTLA4: cytotoxic T lymphocyte associate protein-4
WHO: World Health Organization
MIBC: muscle-invasive bladder cancer
NMIBC: non-muscle invasive bladder cancer
PD-L1: programmed death ligand 1
ICIs: immune checkpoint inhibitors
TMB: tumor mutational burden
ECM: extracellular matrix
TAM: tumor-associated macrophages
TCGA-BLCA: The Cancer Genome Atlas-Bladder Cancer
CNV: copy number variation
OS: overall survival
GSEA: Gene Set Enrichment Analysis
GSVA: Gene set variation analysis
IPS: Immunophenoscore
GDSC: Genomics of Drug Sensitivity in Cancer
IC50: half-maximal inhibitory concentrations
GAPDH: Glyceraldehyde-3-phosphate dehydrogenase
HR: hazard ratios
AUC: Area under the Curve
