# Supplementary figures and images for: Clinical value of M1 macrophage-related genes identification in bladder urothelial carcinoma and in vitro validation
Source: Front Genet. 2022 Nov 16;13:1047004. doi: 10.3389/fgene.2022.1047004 (PMC9709473; doi:10.3389/fgene.2022.1047004)

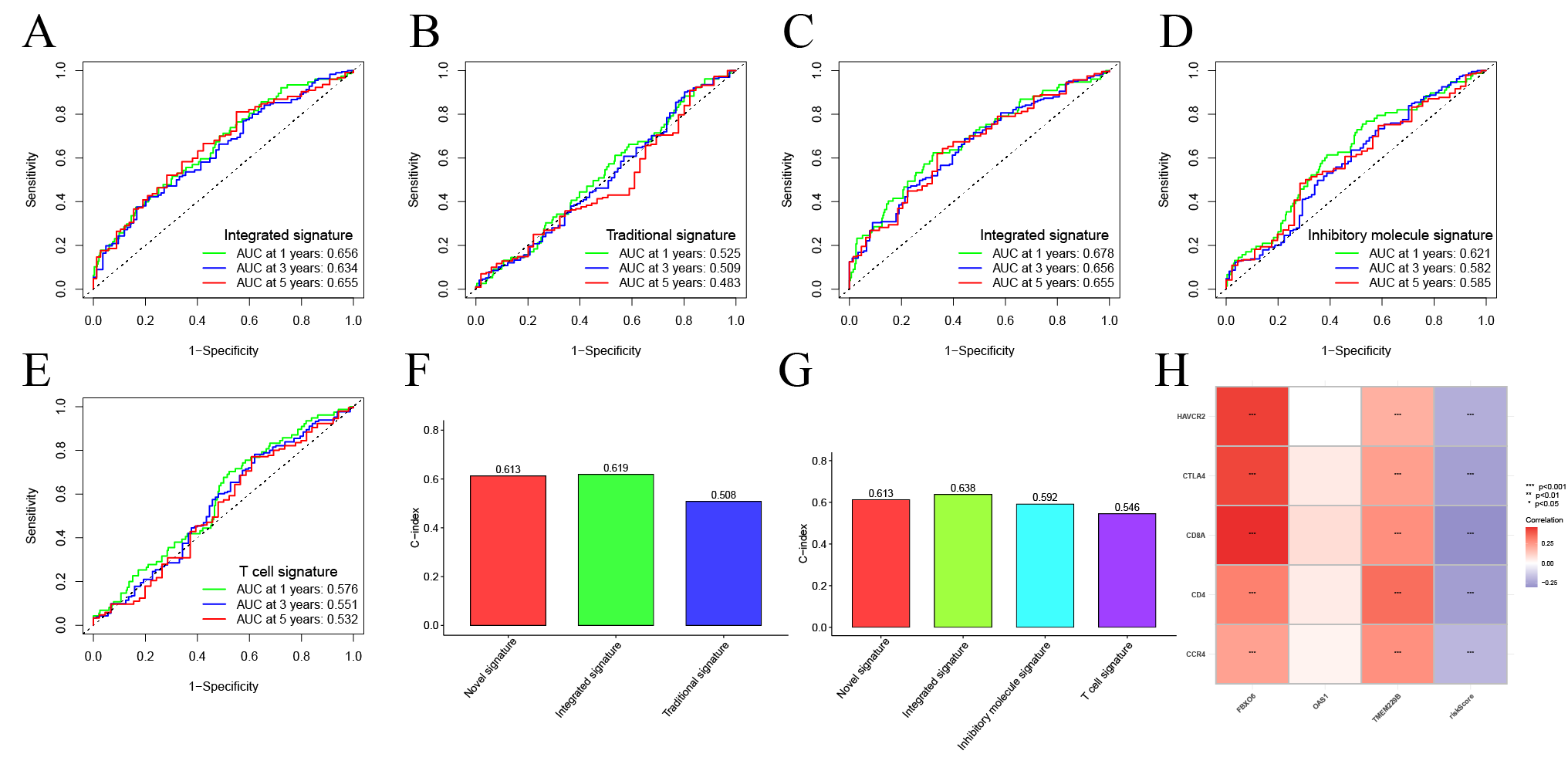

Supplement: Supplementary file 1 [file Image2.TIF]

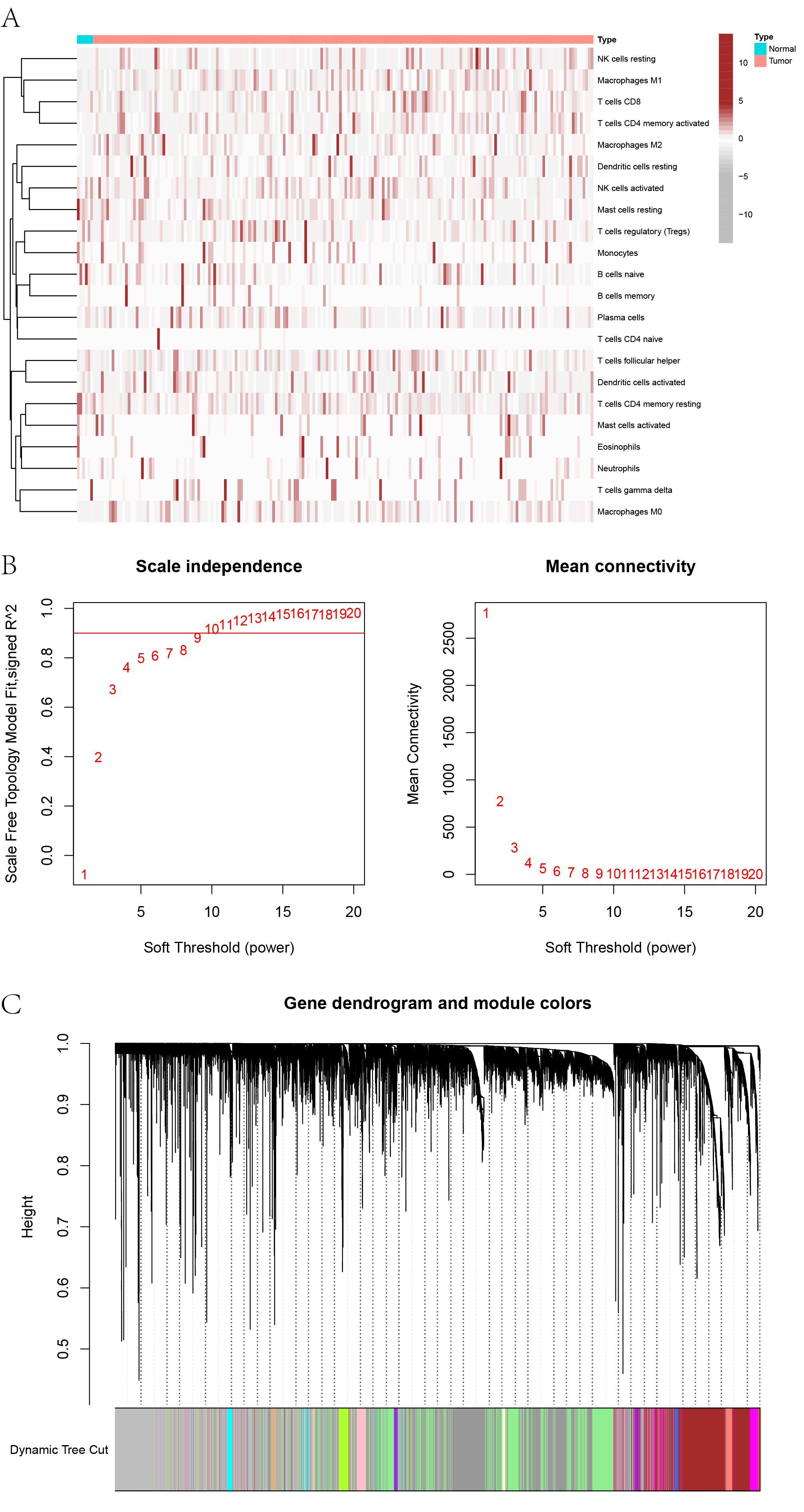

Supplement: Supplementary file 2 [file Image1.TIF]
